# Low-Power Flexible Organic Field-Effect Transistors with Solution-Processable Polymer-Ceramic Nanoparticle Composite Dielectrics

**DOI:** 10.3390/nano10030518

**Published:** 2020-03-12

**Authors:** Xiong Chen, Hao Zhang, Yu Zhang, Xiangfeng Guan, Zitong Zhang, Dagui Chen

**Affiliations:** 1 Organic Optoelectronics Research Center in Fujian Universities, College of Electronics and Information Science, Fujian Jiangxia University, Fuzhou 350108, China; chenxiong@fjjxu.edu.cn (X.C.); zhangh@fjjxu.edu.cn (H.Z.); zhangyu@fjjxu.edu.cn (Y.Z.); xfguan@fjjxu.edu.cn (X.G.); balalapowerzzt@outlook.com (Z.Z.); 2 College of Materials Science and Engineering, Shanghai University, Shanghai 200444, China; 3 College of Physics and Information Engineering, Fuzhou University, Fuzhou 350108, China

**Keywords:** organic field effect transistors (OFETs), calcium titanate nanoparticles, dielectric, solution-process

## Abstract

Polymer-ceramic dielectric composites have been of great interest because they combine the processability of polymers with the desired dielectric properties of the ceramics. We fabricated a low voltage-operated flexible organic field-effect transistor (OFET) based on crosslinked poly (4-vinyl phenol) (PVP) polymer blended with novel ceramic calcium titanate nanoparticles (CaTiO_3_ NPs) as gate dielectric. To reduce interface roughness caused by nanoparticles, it was further coated with a very thin PVP film. The resulting OFET exhibited much lower operated voltage (reducing from –10.5 V to –2.9 V), a relatively steeper threshold slope (~0.8 V/dec) than those containing a pure PVP dielectric. This is ascribed to the high capacitance of the CaTiO_3_ NP-filled PVP insulator, and its smoother and hydrophobic dielectric surface proved by atomic force microscopy (AFM) and a water contact angle test. We also evaluated the transistor properties in a compressed state. The corresponding device had no significant degradation in performance when bending at various diameters. In particular, it operated well continuously for 120 hours during a constant bending stress. We believe that this technology will be instrumental in the development of future flexible and printed electronic applications.

## 1. Introduction

Given the emergence of electronics on skin/organs, rollable displays, printed radio-frequency identification (RFID) tags, electronic papers and wearable sensor, as well as the printability and potential in realizing low-cost and large-area electronic devices, devices with flexible and stretchable organic field-effect transistors (OFETs) have attracted significant attention in the development of next-generation thin-film electronics [1,2].

In fact, one of the major challenges for OFETs has been the rather high voltages needed for their operation when the conventional SiO_2_ is used as the gate dielectrics, which is considered to suffer from its low dielectric constant (k~3.2) [3]. In order to reduce the operating voltage of OFETs, it is necessary to use a high k material and/or to reduce the thickness of the gate dielectric layer [4,5]. However, when the SiO_2_ thickness is reduced to a nanoscale, the gate leakage current derived for the tunneling effect increases exponentially [6]. Although some high-k metal oxide can achieve higher current switch and migration rate, the vacuum and high temperature conditions which are needed for the preparation of inorganic dielectric layer greatly increase manufacturing costs and are unsuitable for flexible plastic substrates. Polymer dielectrics usually afford excellent mechanical flexibility and are compatible with inexpensive plastic foils since they can be processed by coating or printing techniques at low temperatures, but polymers generally have low k, which makes it difficult to meet the requirements for high-density charge storage [7,8,9]. Therefore, the growing appeal in new dielectric materials has arisen primarily from the necessity for an inexpensive device fabrication process and the reduction of the operating voltages required for new flexible/printed electronic technologies [10,11,12].

Polymer/ceramic nanoparticle composite materials have been considered one of the most promising materials for capacitors, since they generally have outstanding electronic properties deriving from the synergy between the two phases, that is the high dielectric permittivity of pure ceramic hosts and the good mechanical strength and ease of film casting of polymer materials. The combination of a solution-processable high dielectric constant BaTiO_3_ or BaSrTiO_3_ nanoparticle layer and other polymers for low-operated organic electronics were reported [13,14,15]. Calcium titanate (CaTiO_3_) ceramics are known as an excellent insulating material with high dielectric constant (k~150) [16], however, no full study of their role as the gate dielectric has been carried out, particularly for flexible electronic applications. 

In this work, we describe the synthesis of ceramic material calcium titanate nanoparticles (CaTiO_3_ NPs) and introduce the novel CaTiO_3_ NPs into crosslinked poly(4-vinyl phenol) (PVP) polymer via simple solution-processing methods. Furthermore, by spinning an additional thin film of PVP, this provides a suitable surface characteristic with a low surface roughness and appropriate surface energy for semiconductors and high dielectric strengths that minimize the leakage current through the gate insulator. Consequently, we fabricate low-voltage operated flexible OFETs with good performance and stability.

## 2. Materials and Methods

### 2.1. Materials Synthetic

Calcium titanate nanopowder is made in the lab. We took 3 mL glacial acetic acid in the high-pressure reaction kettle, and then dissolved butyl titanate (0.3 mL), distilled water (3 mL), CaCl_2_ solution (1 mL, 1 mol/L), an appropriate amount of PVA solution (4 g/L) and NaOH solution (5 mol/L) in the reaction kettle under stirring. We kept adding distilled water until total volume reached 40 ml. We sealed it after stirring evenly, and put it in an oven for chemical reaction at room temperature for a period of time as a subsequent step. The precipitate produced by the above steps was centrifuged and dried at 60℃ to obtain the CaTiO_3_ nanopowder sample.

PVP (polyvinyl phenol, Mw~25,000) with 20 wt% and poly (melamine-co-fomaldehyde) (MMF) with 10% were dissolved in propylene glycol monomethyoether acetate (PGMEA) for use. The MMF was herein used as the cross linker of PVP to remove the hydroxyl groups of PVP. After 4 hours of magnetic stirring at room temperature, CaTiO_3_ nanopowder (3 wt%) was mixed with PVP solution and treated with ultrasonic waves at room temperature for 10 hours to make the particles fully disperse evenly. In order to remove agglomerated particles, the solution was centrifuged at 3000 r/min for 10 min. After that, the precipitate was removed to obtain the PVP: CaTiO_3_ NPs mixture solution. The sample solution for experiments was subsequently filtered with a PTFE filter (0.22 um aperture) to exclude a few remaining larger particles.

Glacial acetic acid, butyl titanate, CaCl_2_, NaOH were received from Aladdin (Shanghai, China). PVA, PVP, MMF, PGMEA were received from Sigma Aldrich (Beijing, China), distilled water was made by Milli-Q Direct16.

### 2.2. Device Fabrication 

**Figure 1:**
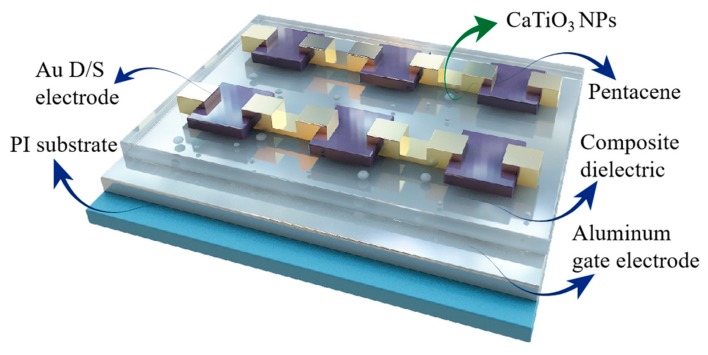
Schematic structures of fabricated organic field-effect transistor (OFET) arrays.

The schematic structure of the fabricated OFET devices is shown in Figure 1. They have a bottom-gate, top-contact structure fabricated on a PI (polyimide) substrate. Initially, 100 nm thick aluminum (Al) serving as the gate electrode were deposited by thermal evaporation on the PI substrate which was treated with ultraviolet (UV) ozone for 5 min; ~300 nm thick film of PVP solution and PVP:CaTiO_3_ nanoparticles mixture solution were deposited on different substrates by spin coating respectively, followed by an annealing process at 120 ℃ for 4 h to produce thermal-crosslinking. For comparison, a thin film of PVP(~50 nm) was additionally spin-coated onto the PVP:CaTiO_3_ nanocomposites to optimize the dielectric layer. 

Afterward, the n-type pentacene (≥99.995% trace metals basis, Sigma Aldrich, Shanghai, China) (~40 nm) was then deposited at a rate of approximate 1 Å/s under a background pressure of 10^−4^ Pa. Gold (Au) (~50 nm) source and drain contacts were deposited by thermal evaporation through a shadow mask. The width and length of the device channel were defined as 900 and 90 μm, respectively. 

### 2.3. Characterization

Crystal phase structures of the CaTiO_3_ nanopowder and pentacene were characterized by an MiniFlex 600 X-ray diffraction (XRD) instrument (Rigagu, TKY, Japan) over the 2θ of 10°to 80°. Electrical characteristics (capacitance vs. voltage, current vs. voltage) of OFETs were performed by a Keithley 4200-SCS semiconductor parameter analyzer (Tektronix, Johnston, OHIO, USA), whereby all electrical measurements were carried out in air. The surface morphologies of the dielectric films and pentacene were characterized with an SPI 400 atomic force microscope (HITACHI, TKY, Japan) in tapping mode. The surface energies of insulators were established by measuring the contact angle using a Kino SL200 KS goniometer. Film thickness was tested by Tencor D-100 step profiler (KLA, Milpitas, CA, USA). During the interval of the bending test, the device is kept bent (50 mm) and placed in a glove box filled with nitrogen to avoid the influence of water and oxygen in the air on the pentacene.

## 3. Results and Discussion

No heterophase diffraction peaks were found in the XRD pattern of calcium titanate (Figure S1), indicating that the nanometer powder prepared in the lab presents a typically pure phase. The mixed solution was stationary for 1 week without precipitation (Figure S1, inset), CaTiO_3_ NPs can be dispersed well in cross-linking PVP solution with uniform size. 

**Figure 2:**
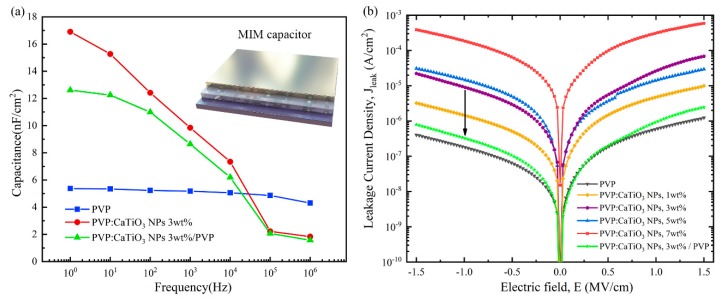
(**a**) Comparison of the frequency-dependent areal capacitance of different dielectric films; (**b**) Leakage current density−electric field characteristics of dielectrics as a function of the CaTiO_3_ nanoparticles (NPs) ratio.

In an OFETs device, the charge density (Q = CV) localized at the first few semiconductor monolayers close to the interface is proportional to the dielectric constant [11,17]. Figure 2 illustrates a parallel-plate metal–insulator–metal (MIM) capacitor that is used to measure the dielectric properties of materials. Figure 2a presents representative frequency-dependent areal capacitance curves for a high-k and a low-k dielectric [8]. It indicates that the addition of calcium titanate ceramic nanoparticles increases the capacity of the dielectric layer. It falls significantly at higher frequencies in the PVP:CaTiO_3_ NPs and PVP:CaTiO_3_ NPs/PVP devicea, since the polarization behavior of high-k materials is different in various frequency ranges, and some polarization cannot follow the change of the high-frequency electric field [18]. The dielectric properties containing PVP layers filled with various concentrations of CaTiO_3_ NPs are shown in Figure 2b, the leakage current density increases as the proportion of CaTiO_3_ NPs increases, which is attributed to the gap between the nanoparticles and the polymer. Due to the modification of thin PVP, the leakage of the capacitor is obviously improved.

**Figure 3:**
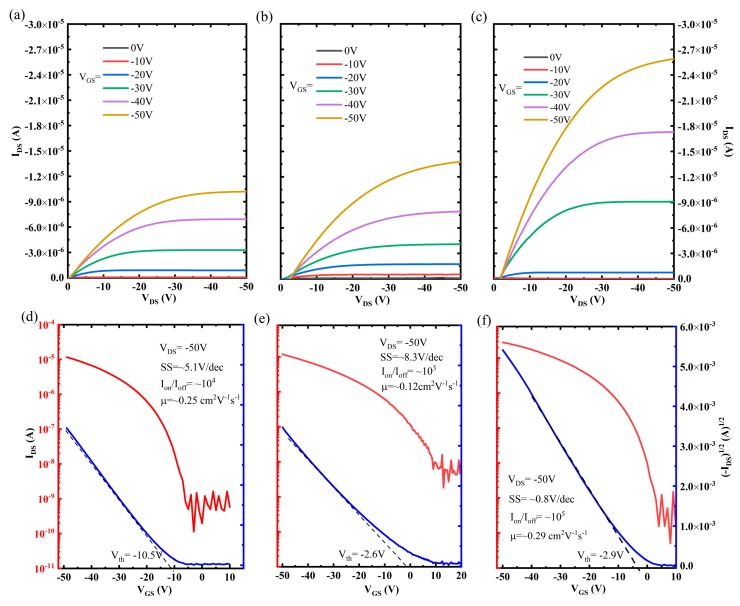
The output and transfer curves of the OFETs with (**a**,**d**) poly (4-vinyl phenol) (PVP), (b,**e**) PVP:CaTiO_3_ NPs, (**c**,**f**) PVP:CaTiO_3_ NPs (3 wt%)/PVP dielectric, respectively. (**a**–**c**) and (**d**–**f**) are in the same scale, respectively.

The electrical performances of OFETs based on various dielectrics are investigated in sequence. The output and transfer characteristics of the devices are presented in Figure 3. It can be seen that the electrical properties of the devices based on different dielectrics vary significantly, including threshold voltage (V_th_), on-off ratio (I_on_/I_off_) and subthreshold slope (SS). The detailed electrical parameters of three types of OFETs are listed in Table 1. The drain current of device composed of PVP insulator filled with 3 wt% CaTiO_3_ NPs is higher than that of devices with a pure PVP dielectric layer. It can be attributed to the large two-dimensional charge density induced in the transistor channel as a result of the high capacitance of the CaTiO_3_ NPs-filled PVP insulator. The more the charge carriers are accumulated at the interface between organic semiconductor and dielectric, the easier the channel could be got through at a lower voltage [19]. Therefore, the use of high-k CaTiO_3_ NPs causes high density charge carriers that result in the reduced V_th_ from –10.5 V to –2.9 V. However, due to the addition of nanoparticles, the gap between the particles and the polymer leads to the increase of leakage current, and the hysteresis is serious (I_on_/I_off_ = ~10^3^, SS = 8.3 V/dec). After being modified by thin PVP, the related performances are dramatically enhanced, achieving the highest I_DS_ and favorable I_on_/I_off_ value which gains an increase of nearly two orders of magnitude, and its leakage current reverts to a lower current (Figure S2). Moreover, the modified device obviously promotes its carrier mobility (μ) from 0.12 cm^2^/Vs^−1^ to 0.29 cm^2^/Vs^−1^, and exhibits a relatively steeper threshold slope (SS) of 0.8 V/decade as compared to that of the unmodified devices, which is due to the reduction of the surface states and charge traps at the contact interface [20]. 

**Figure 4:**
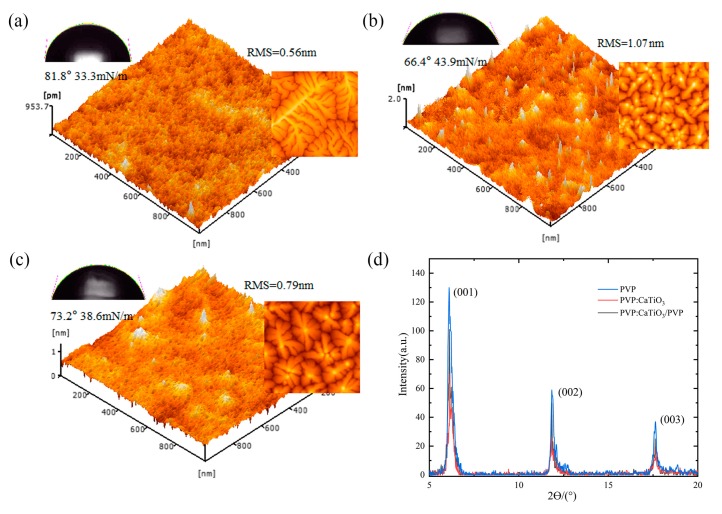
Three-dimensional topography images of (**a**) PVP, (**b**) PVP:CaTiO_3_ NPs, (**c**) PVP:CaTiO_3_ NPs (3 wt%)/PVP dielectric, respectively. Water contact angle (left), atomic force microscope (AFM) image of Pentacene grown on related dielectric. (**d**) X-ray diffraction (XRD) patterns of crystal phase structures of pentacene (40 nm) grown on different dielectrics.

In order to further illustrate the effect of the improved dielectric layer. The test of the AFM and water angel contact is carried out. As shown in Figure 4, the pentacene could grow in an orderly way on flat PVP film with large grain size, while the roughness of the film increases after mixing with high k CaTiO_3_ NPs, resulting in the smaller and discontinuous grain size of the pentacene grown. The increased roughness reduces the carrier mobility of the associated device because of the increased barrier, which limits the hopping of charges in the channel region [21]. After PVP thin-film modification, fewer upheavals can be seen on the surface that lead to the surface roughness and surface energy of the dielectric layer decreasing, reducing from 1.07 nm, 43.9 mN/m to 0.79 nm, 38.6 mN/m, respectively. It gives rise to the increase of the grain size and the decrease of the grain boundary, since the smoother and hydrophobic surface.

XRD tests were performed for the growth of pentacene on different dielectric layers. As depicted in Figure 4d, a sharp diffraction peak appears at 2θ = 5.66°, which corresponds to the (001) crystal plane of pentacene. Based on the data of the (001) diffraction peaks, the average pentacene crystallite sizes were calculated by Scherrer’s formula [22]. They were 42 nm, 25 nm, 35 nm for samples with dielectric of PVP, PVP:CaTiO_3_ NPs, PVP:CaTiO_3_ NPs/PVP, respectively. The diffraction peak intensity of (001) direction of pentacene grown on the surface of PVP:CaTiO_3_ NPs film was lowest, and the corresponding grain size was smallest. After being coated with a thin PVP layer, diffraction peak intensity increased, achieving the larger pentacene. 

**Figure 5:**
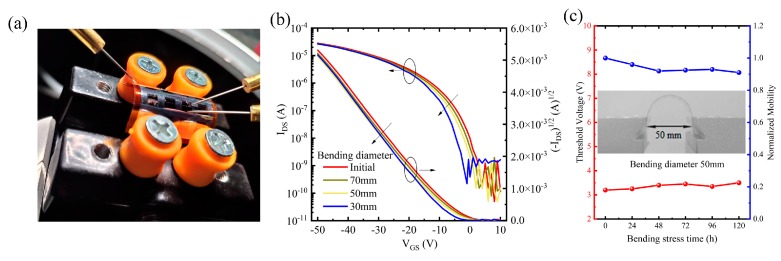
(**a**) Flexible OFETs based on PVP:CaTiO_3_ NPs/PVP composite dielectric with a PI substrate during a bending test. (**b**) Transfer characteristics of Pentacene OFETs based on PVP:CaTiO_3_ NPs/PVP composite dielectric when bending diameter at initial, 70 mm, 50 mm, 30 mm, respectively. (**c**) Evolution of the mobilities and corresponding threshold voltages in OFETs bent at 50 mm under a constant strain.

To investigate the suitability of the device in flexible applications, we evaluated the transistor properties in a compressed state and influence of curvature on device electrical performance; a fixture was used to bend the device and adjust the different curvature to test the electrical performance, as shown in Figure 5a. The distance between the fixtures is defined as the bending diameter (initial, 70 mm, 50 mm, 30 mm, respectively). Figure 5b shows the transfer characteristics of the flexible OFETs devices based on PVP:CaTiO_3_ NPs/PVP composite dielectric when bending at various diameters. As the bending diameter becomes smaller, the threshold voltage of the device is shifted to the negative direction of the X-axis, from –2.9 V to –3.2 V in bending diameter at initial, 70 mm, 50 mm, repectively. The corresponding current on/off ratio (I_on_/I_off_) has negligible change (~10^5^). While it is bent at 30 mm diameter, V_th_ obviously increases to –6.1 V, and current on-off ratio worsens. Particularly, the devices could not function at the further bending compression (10 mm) because of the current breakdown. This is ascribed to the fact that bending widens the spacing between pentacene molecules, where carrier transmission is blocked. Accordingly, V_th_ increases. While applying excessive bending pressure, the related device is subjected to a widening gap between the polymer and ceramic nanoparticles, resulting in a sharp increase in leakage current through the gate.

Moreover, the effects of the mechanical stress on the OFETs characteristics were examined by continuously bending stress for 120 hours at a bending diameter of 50 mm (see in Figure 5c and inset). It can be seen that the changes in mobility and V_th_ in the linear region are negligible during a constant bending stress, indicating good bending stability. 

## 4. Conclusions

In summary, a simple solution process method combining polymer with novel ceramic nanoparticles was proposed for the fabrication of high-performance OFETs based on PI flexible substrates. We have demonstrated that the OFET devices containing CaTiO_3_ NPs in PVP dielectric exhibited a higher field-induced current than those containing a pure PVP due to the increase in the capacitance of the dielectric layer. In order to reduce the increase of interface roughness caused by blending nanoparticles, a very thin PVP film was coated. The result indicates that the modified OFETs with polymer nanoparticle composite dielectric devices operated at a much lower voltage, reducing from –10.5 V to –2.9 V, and improved current on/off ratio to10^5^, relative to conventionally prepared pure PVP-based devices. Moreover, although the addition of nanoparticles increases the capacitance of the dielectric layer so that the threshold voltage of the device is reduced, the surface roughness on the resulting dielectric and the defect state density is increased, which leads to a decrease of the carrier mobility. The modified device promotes its carrier mobility (μ) from 0.12 cm^2^ V^−1^ s^−1^ to 0.29 cm^2^ V^−1^ s^−1^ due to the reduction of the surface states and charge traps at the contact interface. Meanwhile, we tested the effects of the mechanical stress on the OFETs, and it is worth mentioning that there is no mobility and V_th_ degradation for the resulting device after 120 hours while using a continuous bending test at a diameter of 50 mm, indicating that it is mechanically stable upon deformation. This technique provides an effective way to fabricated high quality dielectric and offers great potential for low-cost, fast, and portable organic electronics application.

## Figures and Tables

**Table 1:** Performance criteria of organic field-effect transistors (OFETs) with different dielectrics.

Dielectric Materials	Thickness (nm)	C_i_ (nF/cm^2^) (f = 1 kHz)	k	V_th_(V)	I_on_/I_off_	SS (V/decade)
PVP	300	5.2	1.8	10.5	~10^4^	5.1
PVP:CaTiO_3_ NPs	300	9.9	3.4	−2.5	~10^3^	8.3
PVP:CaTiO_3_ NPs /PVP	350	8.4	3.3	−2.9	~10^5^	0.8

## Supplementary Materials

The following are available online at https://www.mdpi.com/2079-4991/10/3/518/s1, Figure S1: X-ray diffraction pattern of calcium titanate nanopowder. Figure S2: Leakage current of related OFETs device based on different dielectrics.
