# Supplementary material for: Low-Power Flexible Organic Field-Effect Transistors with Solution-Processable Polymer-Ceramic Nanoparticle Composite Dielectrics
Source: Nanomaterials (Basel). 2020 Mar 12;10(3):518. doi: 10.3390/nano10030518 (PMC7153480; doi:10.3390/nano10030518)
Supplement: Supplementary file 1 [file nanomaterials-10-00518-s001.pdf]

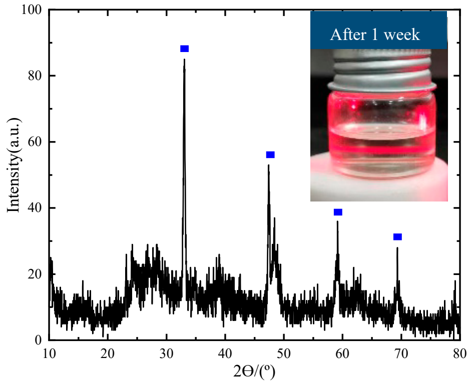

Figure S1. X-ray diffraction pattern of $CaTiO_3$ nanopowder and PVP and $CaTiO_3$ NPs mixed solution (inset).

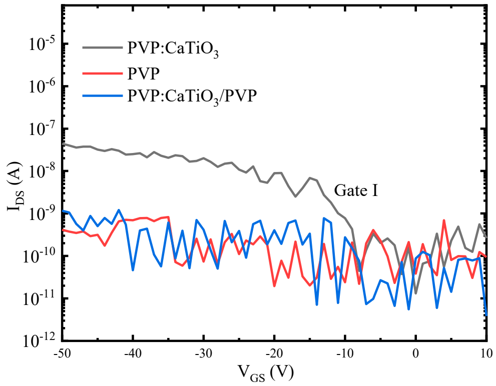

Figure S2. Leakage current of related OFETs device based on different dielectrics..
